# Supplementary material for: Lung Transplantation Outcomes in Recipients Aged 70 Years or Older and the Impact of Center Volume
Source: J Clin Med. 2023 Aug 18;12(16):5372. doi: 10.3390/jcm12165372 (PMC10455483; doi:10.3390/jcm12165372)
Supplement: Supplementary file 1 [file jcm-12-05372-s001.zip › jcm-2517905-supplementary.pdf]

Supplemental Table S1. Generalized linear mixed model coefficients.

	**b**	**se**	**p**
Intercept	-2.62	0.09	< 0.001
Transplant Year	0.09	0.01	< 0.001
Transplant Volume	0.01	0.001	< 0.001
Year * Volume	-0.0009	0.0001	< 0.001

Transplant year and volume were centered prior to analysis and creation of the interaction term.
